# Spatial and temporal dynamics of urban heat environment at the township scale: A case study in Jinan city, China

**DOI:** 10.1371/journal.pone.0307711

**Published:** 2024-09-16

**Authors:** Dongchao Wang, Jianfei Cao, Baolei Zhang, Kangning Kong, Run Wang

**Affiliations:** 1 College of Geography and Environment, Shandong Normal University, Jinan, Shandong, China; 2 Shandong Provincial Territorial Spatial Ecological Restoration Center, Jinan, Shandong, China; National Central University, TAIWAN

## Abstract

The prolonged dependence on industrial development has accentuated the cumulative effects of pollutants. Simultaneously, influenced by land construction activities and green space depletion, the Urban Heat Island (UHI) effect in cities has intensified year by year, jeopardizing the foundation of sustainable urban development. Prudent urban spatial planning holds the potential to robustly ameliorate the persistent deterioration of the UHI phenomenon. This study selects Jinan City as a case study and employs spatial autocorrelation and spatial regression algorithms to explore the spatiotemporal evolution of urban-rural patterns at the township scale. The aim is to identify key factors driving the spatiotemporal differentiation of Land Surface Temperature (LST) from 2013 to 2022. The research reveals a trend of initially rising and subsequently falling LST in various townships, with low-temperature concentration areas in the southern mountainous region and the northern plain area. The "West-Central-East" main urban axis and the southeast Laiwu District exhibit high-temperature zones. Significant influences on LST are attributed to pollution levels, topographical factors, urbanization levels, and urban greenness. The global Moran’s Index for LST exceeds 0.7, indicating a strong positive spatial correlation. Cluster analysis results indicate High-High (HH) clustering in the central Shizhong District and Low-Low (LL) clustering in the northern Shanghe County. Multiscale Geographically Weighted Regression (MGWR) outperforms Geographically Weighted Regression (GWR) and Ordinary Linear Regression (OLR), providing a more accurate reflection of the regression relationships between variables. By investigating the spatiotemporal evolution of LST and its driving factors at the township scale, this study contributes insights for future urban planning and sustainable development.

## Introduction

The intricate interplay of urban ecological environments and economic activities complicates the interpretation of urban spatial patterns, making them inherently complex and variable [1]. Urban patterns significantly alter pollution levels [2], natural environments [3], land use [4], and population distribution [5]. The rapid expansion of urban areas exacerbates negative resident sentiments [6] and triggers various issues related to transportation [7], environment [8], and development [9]. The escalating scale of economic activities within urban spaces results in a surge in urban population and impermeable surface area, while the proportion of vegetation and water bodies continuously diminishes [10]. These urban development challenges elevate urban Land Surface Temperature (LST), widening the temperature difference between urban and rural areas, thereby reinforcing the exposure risk of the Urban Heat Island (UHI) effect [11, 12]. As urban spatial patterns adjust with increasing urbanization, changes in urban characteristics (pollution levels, land use, architectural forms, and population size) significantly alter, indirectly promoting the increase in regional heat production and absorption capacity [13]. In the context of global warming, population expansion, and rapid urbanization, the UHI effect poses a substantial threat to current living standards and future sustainable development [14]. Studying the spatiotemporal patterns of the urban thermal environment and exploring the impact of urban spatial patterns on the thermal environment are crucial for alleviating the increasingly severe negative effects of the UHI.

LST, obtained through thermal radiation measurement techniques [15], is crucial for understanding the UHI effect [16]. Recent advancements in remote sensing technology have greatly expanded the perspectives and methods in the field of UHI. Scholars use remote sensing technology to identify urban impervious layers, establish regression models between impervious layers and LST, and further measure the intensity of the UHI effect [17]. Alternatively, they divide local climate zones [18] within cities according to different criteria, combine building landscape forms [19], and simulate the correlation between various urban indicators [20] and LST. Advanced machine learning techniques are also applied in the field of LST measurement [21], combined with disciplines such as urban climate, human geography, and urban planning, collectively promoting the research on the urban thermal environment [22].

The resolution and accuracy of remote sensing data are crucial for accurately capturing the spatiotemporal variations in the UHI effect [23]. However, current remote sensing technology still has room for improvement in terms of high resolution and high accuracy [24]. The continuous changes in factors such as urban structure, greenery rate, and land use during the process of urbanization have a significant impact on the formation and development of the UHI effect [25]. Therefore, comprehensive monitoring and analysis using multi-source, multi-temporal remote sensing data are necessary. However, current remote sensing technology faces technical challenges in obtaining long time series data and achieving large-area coverage [26]. While remote sensing technology provides information on LST, it needs to be combined with multidisciplinary data such as meteorology, geology, and ecology to comprehensively and deeply understand the mechanisms and impacts of the UHI effect [27]. In addition, collaborative efforts with urban planning and management are essential directions for future research [28]. In summary, although remote sensing technology has achieved some results in the field of the UHI effect and LST inversion, continuous improvements are needed in data resolution, spatiotemporal monitoring capabilities, and multi-source data fusion to better address environmental issues in the urbanization process and provide scientific support for urban sustainable development [29].

This study unlocks a perspective on urban spatial patterns at the township scale, employing spatial regression statistical models and spatial autocorrelation analysis tool to decipher complex urban spatial feature factors and delineate the spatiotemporal patterns and driving elements of the UHI effect. We quantify the spatiotemporal driving effect of township spatial patterns on urban LST from a new perspective, bridging the gap in the study of urban heat environment at the township scale. The main objectives of this paper are as follows: (1) preprocess spatiotemporal image sequences, use a mono-window algorithm to invert LST, and analyze its spatiotemporal patterns; (2) quantify the spatial dependence and spatial clustering characteristics of LST using spatial autocorrelation methods; (3) utilize geographically weighted spatial regression methods to explore the local spatial relationships between urban characteristics and LST.

## Materials and methods

### Study area

**Fig 1:**
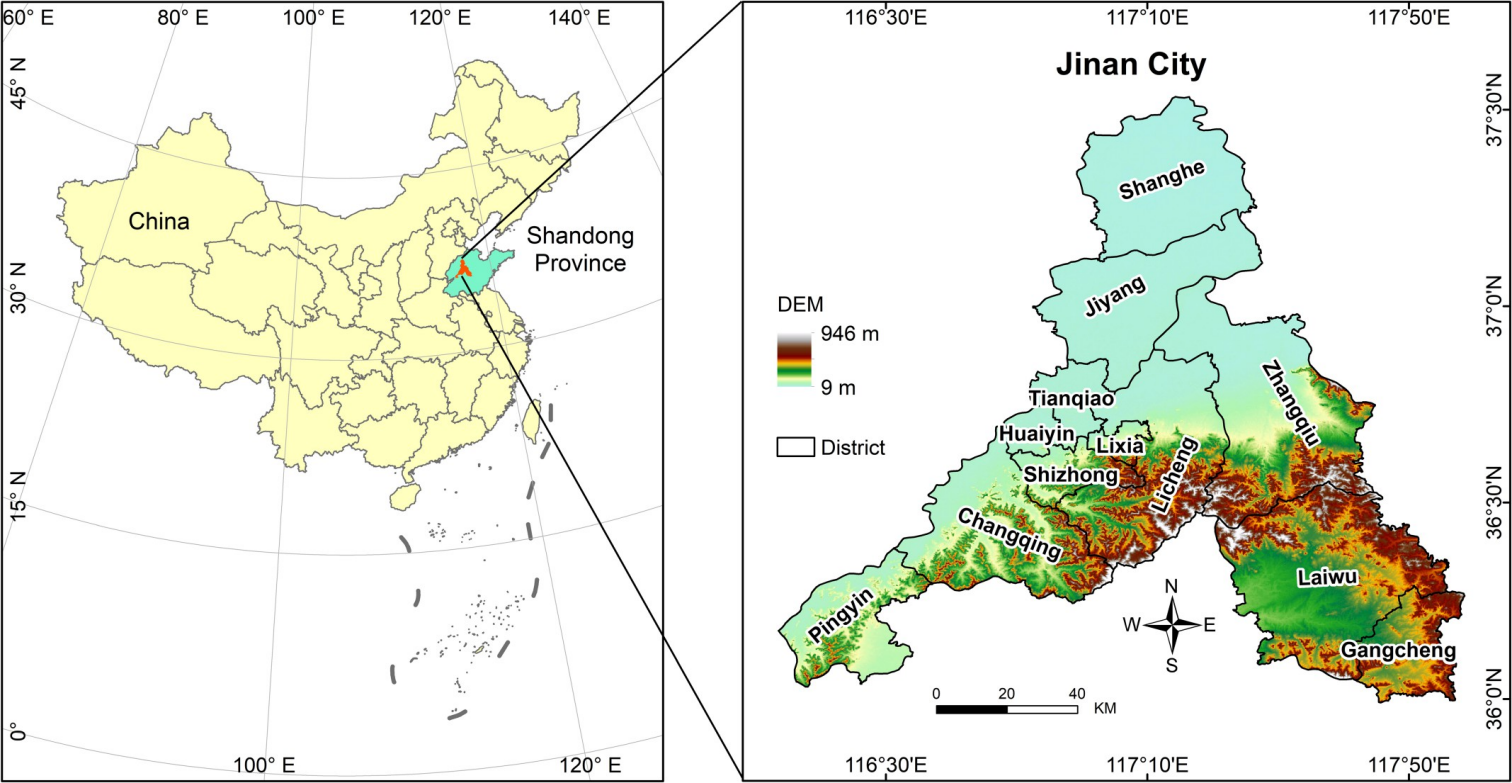
Location and study area (Jinan City, China).

Jinan City is located in the central part of the Shandong Peninsula, with Mount Tai to the south and the Yellow River to the north (Fig 1). Geographically adjacent to the North China Plain, it experiences a typical temperate monsoon climate. Jinan comprises 12 districts and counties, namely, Shizhong (17 townships), Lixia (14 townships), Huaiyin (14 townships), Tianqiao (15 townships), Licheng (21 townships), Changqing (10 townships), Zhangqiu (20 townships), Jiyang (10 townships), Laiwu (15 townships), Gangcheng (5 townships), Pingyin (8 townships), and Shanghe (12 townships). As the capital city of Shandong Province, Jinan possesses substantial political, economic, industrial, and population resources. In recent years, with the continuous enhancement of the city’s overall strength, urban spatial patterns and functional zoning have undergone constant evolution and updates, significantly influencing the UHI effect.

The expanding urban volume continually increases the demand for land and natural resources, leading to intensified pollutant emissions [30]. Townships are crucial administrative units for managing Chinese cities, capable of responding to various strategic urban development plans [31]. This study explores the spatiotemporal patterns of LST and its driving factors at the township scale, providing valuable references for future urban spatial planning.

### Data

#### Data sources

The research dataset includes remote sensing images, digital elevation models, land use data, built-up area information, population data, and urbanization levels (Table 1). MODIS images provide aerosol thickness data for assessing urban air pollution. Landsat images provide surface reflectance data, and a mono-window algorithm is applied for LST inversion. Digital elevation models are obtained from the U.S. Geological Survey (USGS) Earth Resources Observation and Science (EROS) center, covering the entire globe. Land use data utilize the latest 10-meter resolution data released by ESRI (it did not include data prior to 2017, so the data of 2017 were used for 2013 and 2016 in the experiment). Built-up area, population, and urbanization level data are sourced from the Global Human Settlement Layer (GHSL) by the European Commission (EC).

**Table 1:** Data sources.

Data source	Data provider	Spatial resolution	Time
MODIS (MCD19A2v061)	NASA LP DAAC at the USGS EROS Center [32]	1000 m	2013
			2016
			2019
			2022
Landsat 8 OLI/TIRS	USGS EROS Center [33]	30 m	2013
			2016
			2019
			2022
DEM	USGS EROS Center [34]	7.5 arc-second	2010
ESRI 2020 Global Land Use Land Cover from Sentinel-2	Impact Observatory for Esri [35]	10 m	2017
			2019
			2022
GHS built-up surface grid (GHS-BUILT-S)	Global Human Settlement Layer (GHSL), European Commission (EC), Joint Research Centre (JRC) [36]	100 m	2020
GHS built-up height grid (GHS-BUILT-H)	GHSL, EC, JRC [37]	100 m	2018
GHS population grid (GHS-POP)	GHSL, EC, JRC [38]	100 m	2020
GHS urbanisation-degree grid (GHS-SMOD)	GHSL, EC, JRC [39]	1000 m	2020

#### Explanatory variables

**Fig 2:**
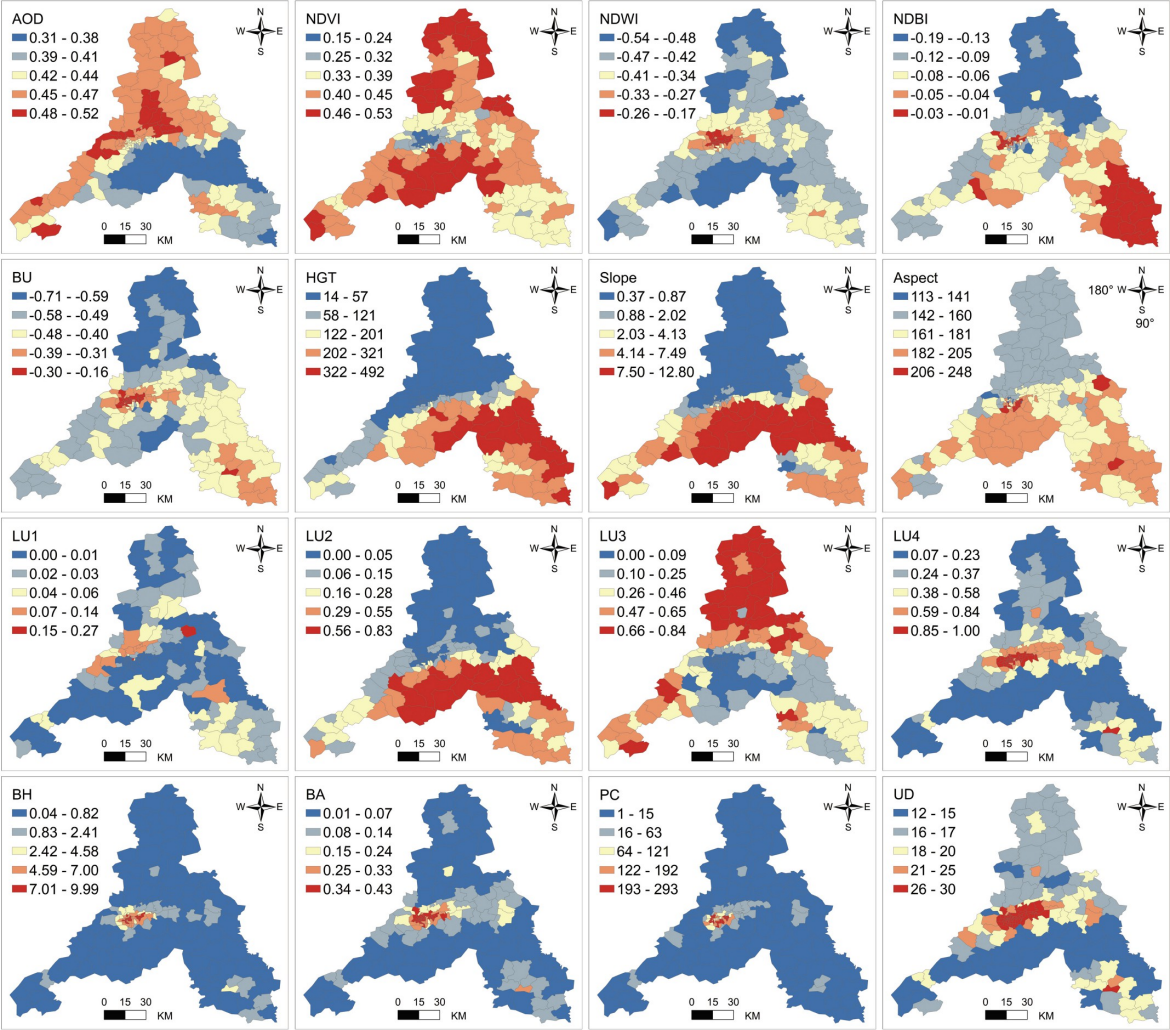
Spatial variations of explanatory variables related to LST.

The relationship between urban thermal environments and urban spatial features is complex, influenced by changes in industry, economy, culture, and public health, all impacting the distribution of urban LST. To study the driving factors of the UHI effect, various types of factors need comprehensive consideration [40]. This study collects six types of factors: Environmental condition, Remote sensing indicators, Terrain, Land use, Building scale, and Socioeconomic factors, with a total of 16 variables (Table 2) [41–44]. Fig 2 illustrates the basic spatial distribution characteristics of explanatory variables at the township scale.

**Table 2:** Variable explanations.

Type	Variable	Description	Formula
Environmental condition	Aerosol Optical Depth (AOD)	Average of AOD per township	—
Remote sensing indicators	Normalized Difference Vegetation Index (NDVI)	Average of NDVI per township	(B_nir_ ‐ B_red_) / (B_nir_ + B_red_)
	Normalized Difference Water Index (NDWI)	Average of NDWI per township	(B_green_ ‐ B_nir_) / (B_green_ + B_nir_)
	Normalized Difference Built-up Index (NDBI)	Average of NDBI per township	(B_mir_ ‐ B_nir_) / (B_mir_ + B_nir_)
	Built-up Index (BU)	Average of BU per township	NDBI ‐ NDVI
Terrain	Elevation (HGT)	Average of elevation per township	—
	Slope	Average of slope per township	—
	Aspect	Average of aspect per township	—
Land use	Proportion of water land (LU1)	Water proportion in each township	AREA_w_ / AREA_t_
	Proportion of vegetation land (LU2)	Vegetation proportion in each township	AREA_v_ / AREA_t_
	Proportion of cultivated land (LU3)	Cultivated land proportion in each township	AREA_cl_ / AREA_t_
	Proportion of construction land (LU4)	Construction proportion in each township	AREA_c_ / AREA_t_
Building scale	Building height (BH)	Average of BH per township	—
	Building area (BA)	Average of BA per township	—
Socioeconomic factors	Population count (PC)	Average of PC per township	—
	Urbanisation degree (UD)	Average of UD per township	—

Prior to using these explanatory variables, a pre-detection process is necessary to address significant multicollinearity issues between variables. Table 3 lists the basic statistical information (Mean, Variance, and Standard deviation), correlations (Pearson correlation coefficient), global Moran’s Index, and variance inflation factors (VIF) for each variable. The results indicate that the VIF values for NDVI, NDWI, NDBI, and BU in remote sensing indicators are higher than 10.0, revealing significant multicollinearity. To correct this issue, a logarithmic transformation is applied to all 16 variables, eliminating interference between variables and ensuring the interpretability of regression results.

**Table 3:** Variable statistical description results.

Variable	Mean	Variance	Standard deviation	Pearson correlation coefficient	Global Moran’s Index	VIF	Logarithmized VIF
AOD	0.421	0.002	0.049	0.430	0.121	9.535	7.275
NDVI	0.422	0.017	0.131	-0.304	0.828	17.771	9.154
NDWI	-0.448	0.015	0.123	0.290	0.911	13.451	8.385
NDBI	-0.087	0.009	0.094	-0.031	0.367	13.525	8.426
BU	-0.509	0.038	0.196	0.184	0.826	16.631	8.864
HGT	166.918	27914.053	167.075	-0.383	0.273	8.127	7.203
Slope	4.356	35.114	5.926	-0.368	0.216	9.835	8.965
Aspect	172.126	12064.994	109.841	0.057	0.154	1.929	1.679
LU1	0.040	0.000	0.000	0.017	0.220	4.431	2.171
LU2	0.528	0.000	0.000	-0.360	0.248	8.609	6.092
LU3	0.884	0.000	0.000	0.213	0.734	7.588	4.791
LU4	0.542	0.000	0.000	0.306	0.805	9.166	8.491
BH	0.477	2.107	1.451	0.192	0.875	7.362	6.395
BS	0.132	0.000	0.000	0.282	0.840	9.951	8.519
PC	8.566	1554.950	39.433	0.181	0.915	7.180	4.070
UD	16.277	38.230	6.183	0.315	0.971	8.187	7.872

### Methods

**Fig 3:**
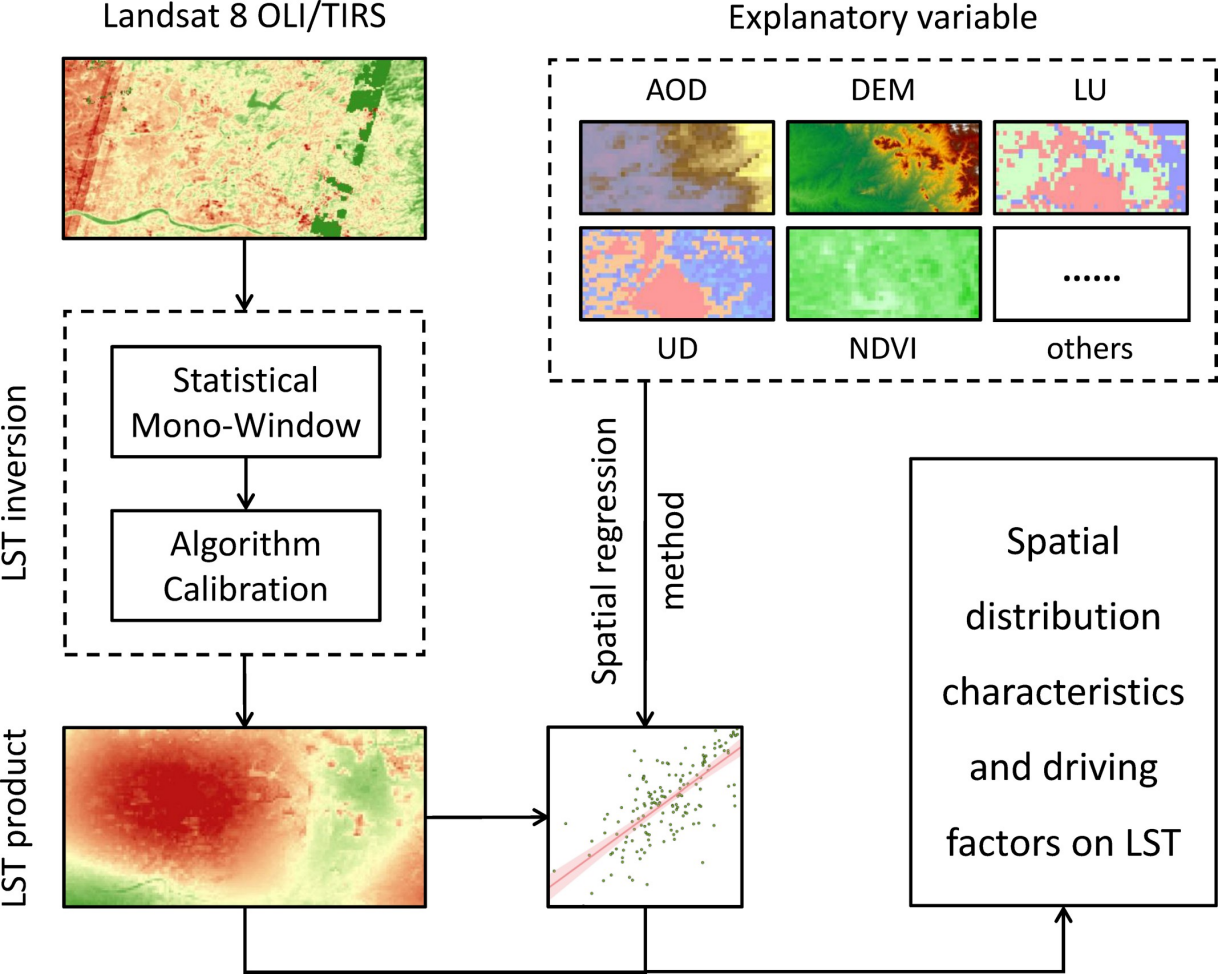
Research technology flowchart.

This study begins at the township scale, using Landsat remote sensing images to invert LST and employing spatial autocorrelation and spatial regression methods to explore the spatiotemporal patterns of LST and its driving factors (Fig 3). First, the statistical mono-window algorithm is utilized to calculate the inversion products of LST based on the radiance brightness temperature and atmospheric parameters derived from satellite images. Then, several explanatory variables related to LST, such as aerosol optical thickness (AOD), digital elevation model (DEM), land use (LU), urbanization degree (UD), normalized difference vegetation index (NDVI), etc., are selected as independent variables for spatial regression analysis. Finally, global (OLR) and local (GWR, and MGWR) regression methods are employed to establish spatial relationship models between LST and explanatory variables, analyzing the spatiotemporal distribution characteristics and driving factors of LST.

#### LST inversion

The Landsat LST standard product often show striped chromatic aberration at mesoscale [45]. The problem is mainly caused by several factors, such as satellite optical system errors, image sensor errors, color processing errors, and the influence of ambient light [46]. It is necessary to repair the strip chromatic aberration present in the data when using it to eliminate the serious impact of data errors on the results of subsequent experiments [47].

The mono-window inversion algorithm for LST is a method used to extract LST information from remote sensing data [48]. Its core idea is to utilize the interaction of thermal radiation observation signals between the atmosphere and the land surface. By establishing a radiative transfer equation, the algorithm inverts the radiance brightness temperature of a single band or window, providing an estimate of LST. In recent years, many researchers have adopted this method for LST inversion to ensure data accuracy and stability [49–51].

In the mono-window algorithm, considerations typically include atmospheric transmittance, absorption, scattering, and emission factors to establish a complex radiative transfer model. By utilizing observed radiance brightness temperature data and mathematical optimization algorithms, model parameters are adjusted to make the model calculation results as consistent as possible with the observed data, obtaining the optimal estimate of LST. The specific algorithm is as follows:

C=ετ (1)

D=(1−τ)[1+(1−ε)τ] (2)

$$L_{\lambda }=scale\;*\;DN+\;offset$$ (3)

$$T_{a}=\frac{K_{2}}{ln\left(1+\frac{K_{1}}{L_{\lambda }}\right)}$$ (4)

$$T_{s}=\frac{\left(a(1-C-D)+T_{a}(b(1-C-D)+C+D)-DT_{b}\right)}{C}-273.15$$ (5)

In the formula, *ε* represents the land surface emissivity; *τ* represents atmospheric transmittance; *K*_*1*_ and *K*_*2*_ are preset constants for Landsat-8, where *K*_*1*_ = 774.89, and *K*_*2*_ = 1321.08; *a* and *b* are fitting coefficients with values -67.355351 and 0.458606, respectively; *C* and *D* are intermediate variables; *L*_*λ*_ represents radiance intensity values, with gain parameter *scale* = 0.0003342, *offset* = 0.1, and digital number value *DN* representing pixel radiance; *T*_*a*_ represents brightness temperature, *T*_*b*_ represents atmospheric average temperature, and *T*_*s*_ is the target LST.

#### Spatial regression models

Geographically Weighted Regression (GWR) is a regression analysis method used for spatial data [52]. Unlike traditional global regression models, GWR allows the model’s parameters to vary spatially, better capturing heterogeneity and local correlations in geographic spatial data [53]. The regression model of GWR can be expressed as:

$$Y_{i}=\beta_{i}^{0}+\sum_{k=1}^{P}\beta_{i}^{k}X_{i}^{k}+\varepsilon_{i}$$ (6)

Where *Y*_*i*_ is the observed value of the dependent variable at geographic location *i*; *P* corresponds to the number of independent variables; $X_{i}^{k}$is the observed value of independent variable *k* at geographic location *i*; $\beta_{i}^{0}$ and $\beta_{i}^{k}$ are the intercept and regression coefficient at geographic location *i*; ε_*i*_ is the error term.

The key point of GWR is the use of different regression coefficients for each geographic location. The spatial weight matrix reflects the variation of spatial dependence within the study area. This allows GWR to have the ability to detect spatial heterogeneity and improve the accuracy of regression fitting. GWR generally uses a specific distance decay function to determine the elements of the weight matrix, and the spatial bandwidth controls the decay rate. Samples closer in distance have larger weights, while those farther away have smaller weights.

Multiscale Geographically Weighted Regression (MGWR) is an improved spatial regression model that can be used to explore multiscale variations in spatial heterogeneity [54]. Compared to GWR, which uses a fixed global bandwidth, MGWR assigns an independent spatial bandwidth for each variable. Therefore, MGWR can flexibly capture spatial heterogeneity of different regression coefficients and more accurately reflect local relationships in spatial data [55]. Moreover, MGWR can consider both global and local information, providing a more flexible balance between global and local factors.

#### Spatial autocorrelation analysis

In urban thermal environment research, spatial autocorrelation is a method used to describe the spatial correlation of geographic data. Moran’s I is a commonly used spatial autocorrelation statistic, measuring the overall spatial correlation degree of geographical data [56]. It can be divided into global Moran’s Index and local Moran’s Index. The calculation formulas are as follows:

$$Global\;Moran's\;I=\frac{n\sum_{i=1}^{n}\sum_{j\neq i}^{n}W_{ij}\left(x_{i}-\bar{x}\right)\left(x_{j}-\bar{x}\right)}{\sum_{i=1}^{n}\sum_{j\neq i}^{n}W_{ij}\sum_{i=1}^{n}{\left(x_{i}-\bar{x}\right)}^{2}}$$ (7)

$$Local\;Moran's\;I=\frac{n\left(x_{i}-\bar{x}\right)\sum_{j=i}^{n}W_{ij}\left(x_{j}-\bar{x}\right)}{\sum_{i=i}^{n}{\left(x_{i}-\bar{x}\right)}^{2}}$$ (8)

Where *n* is the number of geographic units; *x*_*i*_ is the observed value of the *ith* geographic unit; $\bar{x}$is the mean value of all geographic units; *W*_*ij*_ is the spatial weight between geographic unit *i* and geographic unit *j*. The Moran’s Index ranges from -1 to 1, where positive values indicate positive correlation and negative values indicate negative correlation. Values close to 1 or -1 suggest significant spatial clustering patterns.

Local Indicators of Spatial Association (LISA) aggregation analysis is used to detect local spatial clustering patterns in geographic space. By calculating the Moran’s Index between each geographic unit and its neighboring units, geographic units with significant spatial clustering or dispersion patterns can be identified. LISA aggregation analysis produces four quadrants: High-High (HH), Low-Low (LL), High-Low (HL), and Low-High (LH). In the HH and LL quadrants, the observed values of geographic units exhibit clustering patterns, while in the HL and LH quadrants, they show dispersion patterns. This helps understand the local clustering characteristics in spatial distribution.

## Analysis results

### Spatiotemporal patterns of LST

**Fig 4:**
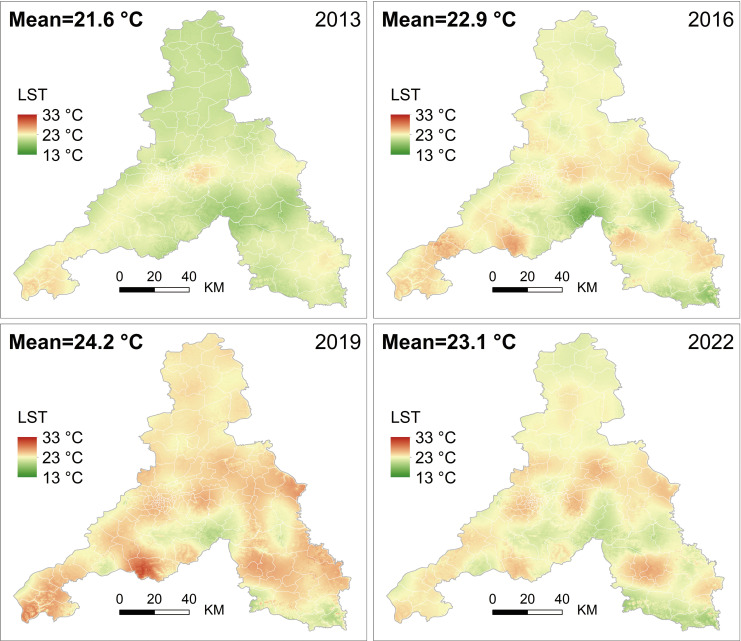
Spatiotemporal distribution characteristics of LST in 2013, 2016, 2019, and 2022.

Utilizing the mono-window inversion algorithm for LST, this study obtained spatiotemporal distribution maps of LST in Jinan for the years 2013–2022 (Fig 4). The daytime annual average LST in Jinan exhibited a continuous upward trend from 2013 to 2019, followed by a decline over the subsequent three years. Originating from the southwest in Pingyin County, the temperature rise extended in a northeastern direction, connecting Changqing District, Huaiyin District, Shizhong District, Tianqiao District, Licheng District, and Lixia District, ultimately spanning the "Western-Central-Eastern" main urban axis. This axis, along with the southeastern Laiwu District, characterized by flat terrain dominated by urban land use, industrial development, and high population density, demonstrated higher LST. In contrast, the southern mountainous regions and the northern plain areas, separated by the Yellow River from the city center, exhibited relatively lower temperatures, primarily featuring forests and farmland.

This study conducted regional statistical analysis on LST raster data (Fig 4), yielding township scale results. In 2013, Tangye Street in Licheng District recorded the highest LST at 24.2°C, while Duozhuang Township in Zhangqiu District exhibited the lowest LST at 19.6°C. In 2016, Ancheng Township in Pingyin County and Xiying Township in Licheng District reported the highest LST at 25.4°C and the lowest LST at 18.1°C, respectively. By 2019, the peak LST shifted to Wande Township in Changqing District, reaching 26.8°C, while Xiying Township in Licheng District maintained the lowest LST at 21.0°C. In 2022, Jiefanglu Street in Lixia District revealed the highest LST at 23.6°C, while Wenyuan Street in Gangcheng District marked the lowest at 20.5°C. Throughout the nine years, Wande Township in Changqing District and Xiying Township in Licheng District documented the highest and lowest LSTs in Jinan, respectively.

**Fig 5:**
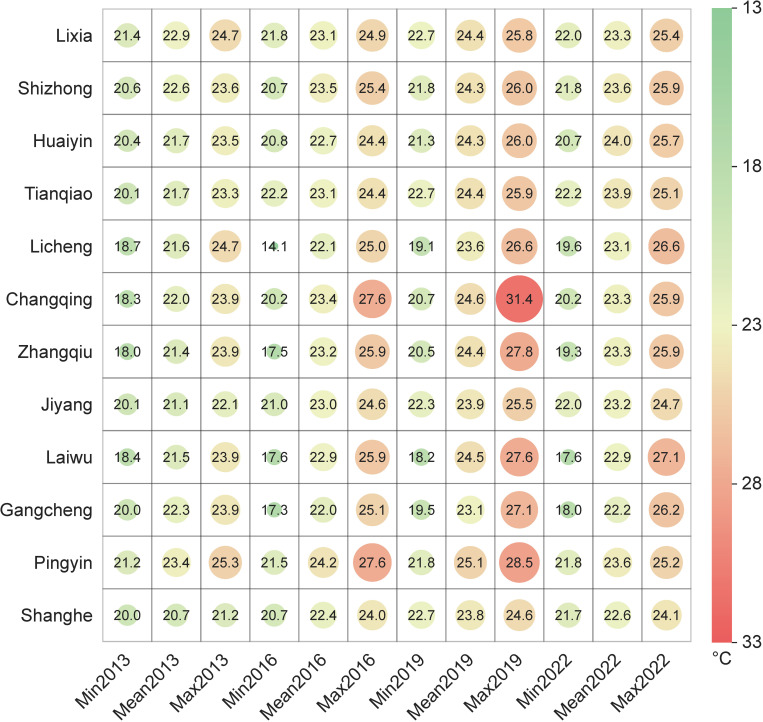
Statistical analysis of LST in counties of Jinan. The numbers show the LST data; the larger the circle radius the higher the LST value; Min, Mean, and Max represent the minimum, mean, and maximum values for each county or district.

Conducting a detailed spatial statistical analysis at the township level for each county in Jinan, Fig 5 illustrates the minimum, mean, and maximum LST for the four periods. Overall, a rising-then-falling temperature trend is observed. Lixia District, Shizhong District, and Pingyin County consistently exhibited higher average temperatures, while Shanghe County and Jiyang District displayed lower values. Examining the highest and lowest temperatures in different years, Changqing District and Pingyin County recorded the highest temperatures of 31.4°C and 28.5°C in 2019, whereas Licheng District and Gangcheng District experienced the lowest temperatures of 14.1°C and 17.3°C in 2016. Notably, Laiwu District, Zhangqiu District, Jiyang District, and Shanghe County demonstrated the most significant average temperature variations, with maximum increases of 3.1°C, 3.1°C, 3.0°C, and 2.8°C, respectively, over the nine-year period.

### LST regression results and influencing factors

To visually compare the regression effects of different models on LST, the OLR, GWR, and MGWR models were successively applied to fit the temperature data. In this experiment, 16 explanatory variables from various townships in 2022 were selected as independent variables, with the inverted LST results serving as the dependent variable. Prior to the regression experiment, logarithmic transformations were applied to all variables to correct data distribution skewness, eliminate multicollinearity among variables, and enhance the explanatory power of the regression model.

Table 4 illustrates the regression analysis results for OLR, GWR, and MGWR models, with evaluation metrics including R2, adjusted R2, root mean square error (RMSE), and Akaike information criterion (AIC). Compared to OLR and GWR, the MGWR model achieved higher R2 and adjusted R2 values, along with lower RMSE and AIC levels. The respective values for MGWR were 0.872, 0.685, 0.521, and 329.840. Clearly, the MGWR model outperformed OLR and GWR in terms of fitting effectiveness, leading to its selection for explaining the regression relationships between variables.

**Table 4:** The regression analysis results for OLR, GWR, and MGWR models.

Type	Variable	OLR	GWR (Mean)	MGWR (Mean)
	Constant	266.461	258.779	257.971
Environmental condition	Log(AOD)	18.670	19.544	7.300
Remote sensing indicators	Log(NDVI)	-579.959	-563.490	-549.528
	Log(NDWI)	10.399	10.073	3.060
	Log(NDBI)	599.257	586.002	580.586
	Log(BU)	-592.151	-577.388	-563.303
Terrain	Log(HGT)	-0.001	-0.001	-0.009
	Log(Slope)	0.072	0.075	0.050
	Log(Aspect)	0.005	0.004	-0.005
Land use	Log(LU1)	-0.660	-0.598	-2.431
	Log(LU2)	0.009	-0.033	-0.150
	Log(LU3)	0.097	0.107	0.389
	Log(LU4)	0.895	1.074	1.509
Building scale	Log(BH)	0.038	0.063	0.064
	Log(BA)	-4.067	-5.087	-8.750
Socioeconomic factors	Log(PC)	0.002	0.002	-0.018
	Log(UD)	0.059	0.067	0.006
	R^2^	0.445	0.550	0.872
	Adjusted R^2^ (R2 adj)	0.383	0.453	0.685
	RMSE	0.774	0.651	0.521
	AIC	408.192	399.205	329.840

### LST spatial autocorrelation analysis

**Fig 6:**
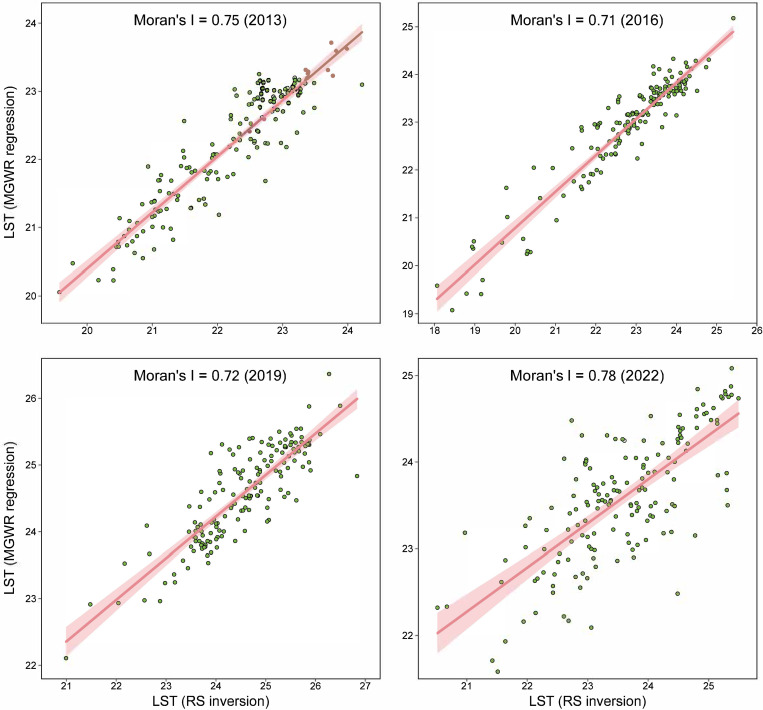
Moran scatter plots of LST in Jinan for various periods.

Global and local spatial autocorrelation analyses were employed to explore the spatial heterogeneity of LST. Fig 6 presents Moran scatter plots illustrating the fitted relationship between remotely sensed LST and MGWR estimations for the four periods, establishing a foundation for quantifying the spatial differentiation of LST data. The results indicate that the global Moran indices for Jinan LST during 2013–2022 were 0.75, 0.71, 0.72, and 0.78, respectively, demonstrating a significant positive spatial correlation.

**Fig 7:**
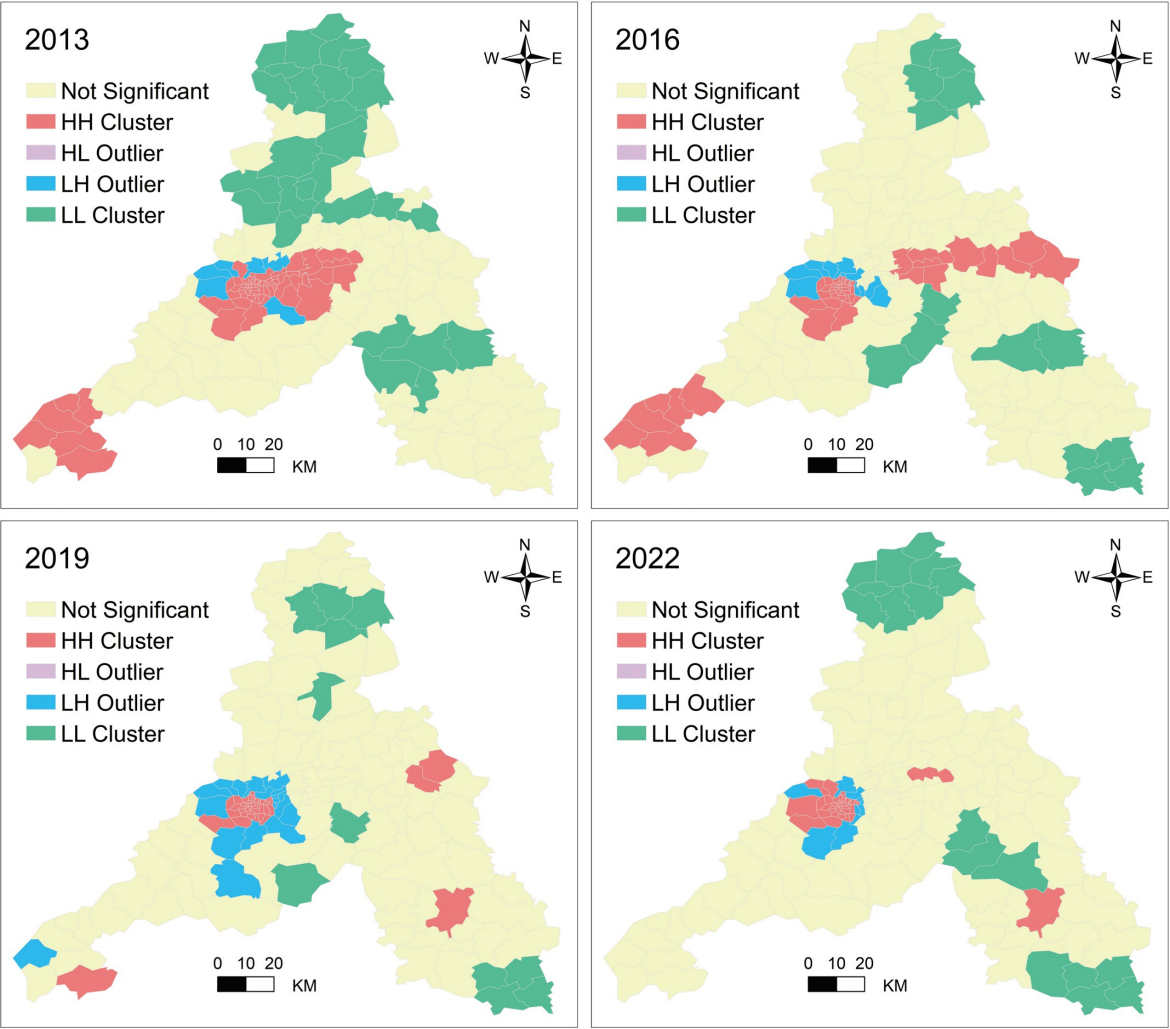
The spatial distribution clustering of LST in the years 2013, 2016, 2019, and 2022.

Fig 7 provides LISA distribution maps obtained through spatial clustering analysis for the four periods. These maps effectively explain the local spatial autocorrelation variations of LST at the township level. The spatial distribution patterns of LST during 2013–2022 generally remained similar. High-high correlation (HH) primarily occurred in the central urban areas, with occasional distribution in Lixia District, Zhangqiu District, Laiwu District, and Pingyin County. Low-low correlation (LL) was concentrated in Shanghe County, with scattered occurrences in Gangcheng District, Zhangqiu District, Laiwu District, and Jiyang District. Low-high correlation (LH) generally surrounded the high-high correlation (HH) areas, geographically close to the city center.

## Discussion

### Driving factors of urban heat island effect

Traditional approaches to uncovering drivers of LST often rely on a singular or a few factors [57], such as local climatic zones, urban landscapes, land use, visible light remote sensing indices, and impervious urban surfaces. However, the impact of the UHI effect extends beyond the city center and may affect remote rural areas or neighboring cities [58]. As the natural hub for residents’ production and life in the field, the normal functioning of urban systems depends on the coordinated interaction of various factors. Changes in air quality, topography, land use, socioeconomic conditions, and remote sensing indicators all contribute to LST in urban areas. Considering as many influencing factors as possible, the derived factors hold more scientific guidance significance.

Table 5 presents the diagnostic results of the correlation between LST and its influencing factors for four consecutive years. It is evident from the table that the environmental factor AOD consistently exhibits the strongest positive correlation with LST (r_2013_ = 0.455, r_2016_ = 0.400, r_2019_ = 0.386, r_2022_ = 0.430; P<0.001). Terrain factors such as HGT and Slope, as well as remote sensing indicators like NDVI, demonstrate a strong negative correlation with LST (r<-0.300; P<0.001). Significant positive correlations (r>0.300; P<0.001) are observed between land use type LU4, socioeconomic UD, and LST. However, building scale such as BH and BS show no significant positive correlation with LST (r<0.290; P<0.001). Based on the correlation analysis results of driving factors, it is determined that environmental conditions, topography, land use types, socioeconomic conditions, and remote sensing indicators are more critical than building scale.

**Table 5:** Correlation analysis between LST and variables in the years 2013, 2016, 2019, and 2022.

Variable	2013		2016		2019		2022	
	r-value	P-value	r-value	P-value	r-value	P-value	r-value	P-value
AOD	0.455	0.0000	0.400	0.0000	0.386	0.0001	0.430	0.0000
NDVI	-0.300	0.0000	-0.304	0.0000	-0.306	0.0000	-0.304	0.0000
NDWI	0.269	0.0000	0.195	0.0000	0.215	0.0000	0.290	0.0000
NDBI	0.278	0.0000	-0.025	0.0000	0.156	0.0000	-0.031	0.0001
BU	0.181	0.0000	0.112	0.0001	0.134	0.0000	0.184	0.0001
HGT	-0.395	0.0001	-0.353	0.0000	-0.350	0.0000	-0.383	0.0000
Slope	-0.359	0.0001	-0.326	0.0000	-0.364	0.0002	-0.368	0.0000
Aspect	0.304	0.0000	0.036	0.0001	0.097	0.0001	0.057	0.0002
LU1	-0.051	0.0002	-0.067	0.0002	-0.048	0.0000	0.017	0.0002
LU2	-0.281	0.0002	-0.304	0.0000	-0.268	0.0001	-0.360	0.0000
LU3	-0.073	0.0004	0.210	0.0000	0.185	0.0000	0.213	0.0000
LU4	0.394	0.0000	0.381	0.0002	0.332	0.0000	0.306	0.0000
BH	0.124	0.0000	0.127	0.0001	0.111	0.0000	0.192	0.0001
BS	0.166	0.0000	0.183	0.0002	0.143	0.0000	0.282	0.0000
PC	0.151	0.0000	0.113	0.0001	0.117	0.0000	0.181	0.0002
UD	0.360	0.0000	0.310	0.0000	0.350	0.0001	0.315	0.0000

**Fig 8:**
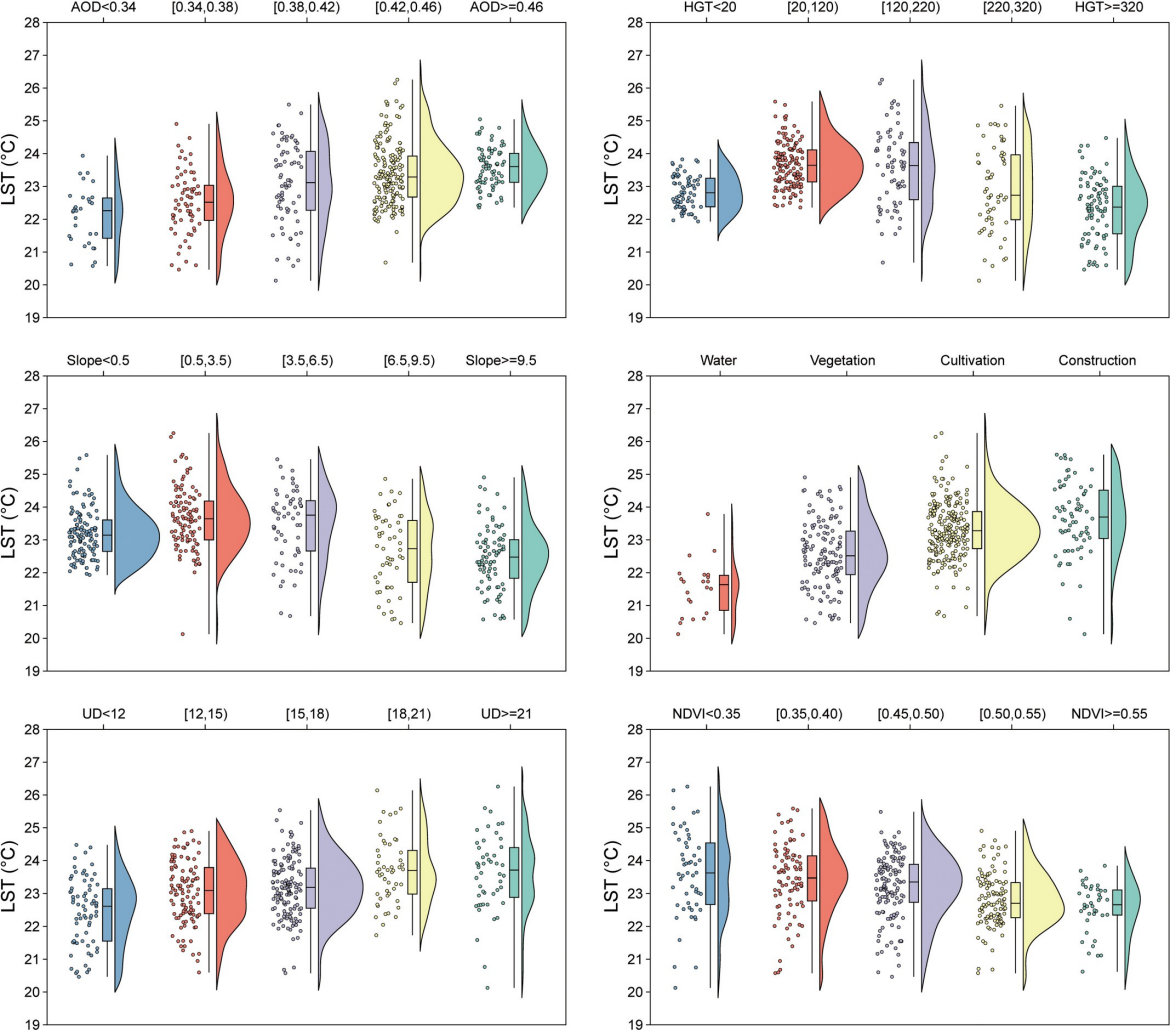
Regulatory effect of warming and cooling factors on LST.

This study conducted grouped statistical analysis of LST based on AOD, HGT, Slope, LU, UD, and NDVI. The results are presented in Fig 8 in the forms of cloud-rain, box, and scatter plots. Land use types include water bodies, vegetation, cropland, and built-up areas. Here, these four land types are amalgamated into subcategories of a single indicator, and their LSTs are uniformly subjected to statistical analysis. AOD is positively correlated with LST. Beyond an altitude of 120 meters, higher HGT is associated with lower LST. As the slope increases, LST decreases. Among land use types, water bodies exhibit the lowest LST, followed by vegetation, cropland, and built-up areas with the highest LST. Larger UD values are associated with higher LST, while NDVI shows a negative correlation with LST. These six factors drive LST variations in different ways, consequently influencing the UHI effect.

### Temporal resilience of urban sustainability

**Fig 9:**
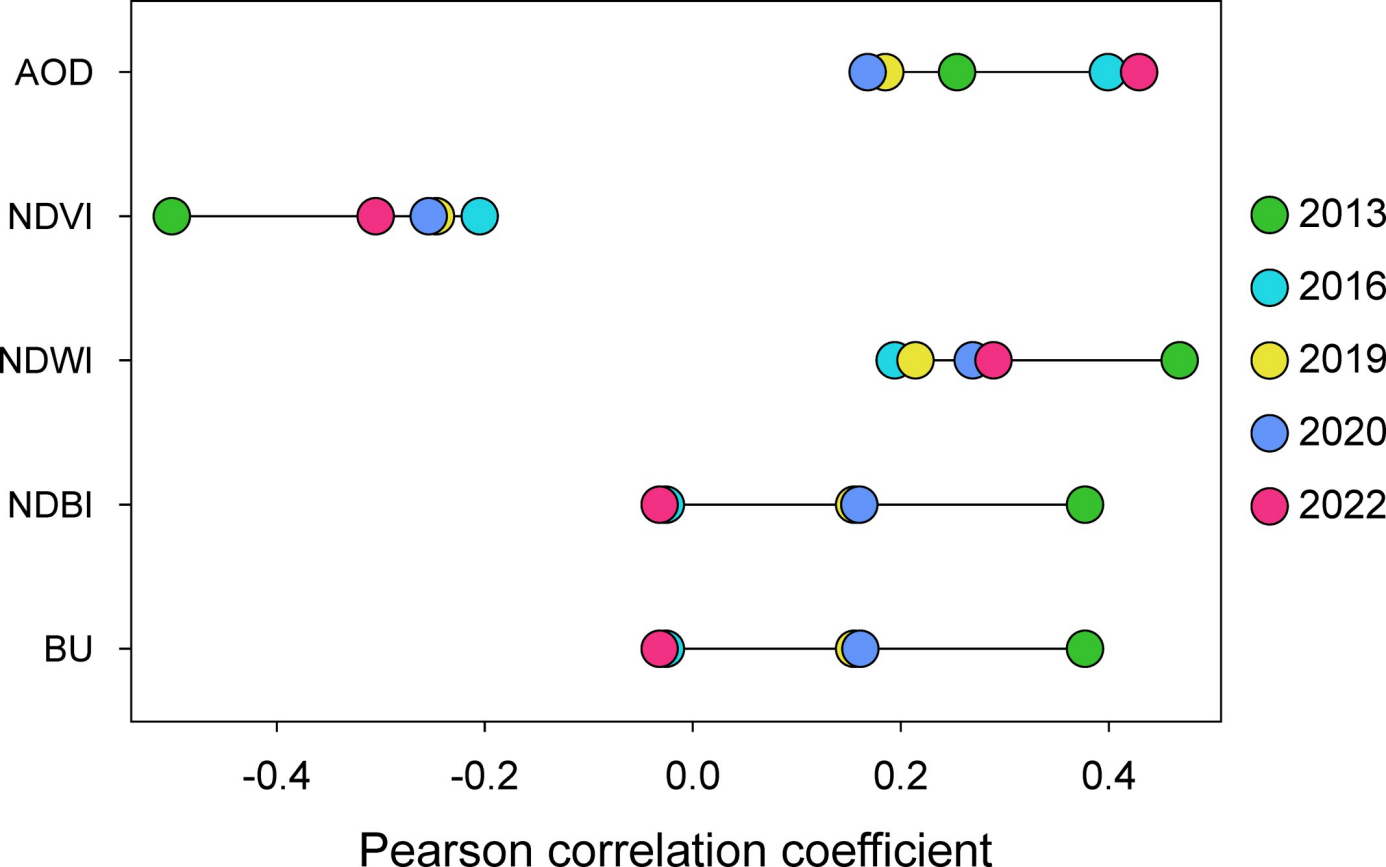
Temporal changes in correlation strength between explanatory variables and LST.

Temporal resilience of urban sustainability pertains to a city’s adaptive capacity over time to various shocks and changes, reflecting the degree of sustainable development in the face of challenges at different periods [59]. Emphasizing the dynamic, adaptive, and responsive aspects of urban sustainability over long-term evolution, temporal resilience underscores the need for cities to maintain stability and development across different periods. As urbanization accelerates, the UHI effect has become a decisive force in urban microclimates [60], progressively impinging on sustainable development. Through a correlation analysis of variables over time, sensitivity factors for LST can be identified. Fig 9 presented in the form of a segmented dumbbell plot, illustrates the changing strength of correlations between explanatory variables (AOD, NDVI, NDWI, NDBI, BU) and LST in 2013, 2016, 2019, 2020 and 2022.

Distinguished from the other four variables, the correlation dynamics of AOD appear unique, registering its lowest value in 2020 but surpassing the 2013 level in 2022. Plausibly, this divergence could be attributed to the significant global events during 2019–2022, particularly the outbreak of the COVID-19 pandemic at the end of 2019 [61]. Research has demonstrated substantial global impacts of the pandemic, including financial market volatility, production disruptions, and increased unemployment. The specific effects varied across countries, regions, and industries. During the pandemic, the reduced economic activity in Jinan likely led to decreased air pollution sources [62], contributing to an improvement in the self-purification ability of the environment. The phenomenon indirectly drives the urban LST fallback event from 2020 to 2022.

### Limitations and future prospects

This study, relying on remote sensing data and other geographical data, employed spatial statistical methods and spatial autocorrelation analysis to explore the spatiotemporal characteristics and driving factors of LST at the township level. Nevertheless, there are several limitations and shortcomings in the content and methodology of the study. (1) The experiment’s acquired LST data represent daytime averages for the entire year, without considering temporal variations such as day-night differences, monthly variations, or seasonal changes. (2) The study exclusively addresses spatial scales at the township level, overlooking potential errors arising from spatial heterogeneity expression at different scales. (3) Due to limitations in data acquisition frequency, the availability of explanatory variables for all periods was constrained. The use of variables from a single period inadequately captures the real-time dynamics of temperature.

Future research should prioritize investigations into multiple time scales, multiple spatial scales, and real-time multi-source data. A comprehensive exploration of the spatiotemporal characteristics and driving factors of LST necessitates the combined application of global statistical methods, local statistical methods, and spatiotemporal statistical methods. A few points need to be noted when applying the research methodology of this study to other regions or scales. (1) The climatic type and intensity of human activities in the target area strongly influence the spatiotemporal distribution patterns of LST driving factors [63], and the degree of correlation will also vary geographically. (2) Different research scales yield different results. District-level analysis is suitable for large areas with sparse population densities [64], while Street-level analysis is more suitable for densely populated areas [65]. The choice of scale should be based on the specific research requirements. In conclusion, researchers need to consider data selection, preprocessing, and research objective setting to eliminate the negative impact of uncertainties on the feasibility of the study.

## Conclusion

This study, employing the classical mono-window algorithm, inverted LST in Jinan city. Utilizing spatial regression statistical models and spatial autocorrelation analysis tool, it delineated the spatiotemporal patterns and driving factors of the UHI effect at the township level. The study’s main findings can be summarized as follows:

1. From 2013 to 2019, LST in various townships gradually increased, followed by a subsequent decline. The southern mountainous regions with dense vegetation and the northern plain areas primarily featuring farmland exhibited lower temperatures. The "Western-Central-Eastern" main urban axis, along with the southeastern Laiwu District characterized by urban land use, industrial development, and high population density, experienced higher LST.
2. During the period of 2013–2022, both global and local Moran indices for LST in Jinan were consistently high, affirming a significant positive spatial correlation. LISA results demonstrated that HH correlation mainly occurred in the city center, while LL correlation was concentrated in the northern regions. LH correlation typically surrounded the HH correlation areas, geographically close to the city center.
3. Factors driving LST are diverse, with environmental conditions exhibiting the strongest correlation, followed by topography and land use types. Subsequently, socioeconomic conditions and remote sensing indicators demonstrated correlation, while the correlation with building scale was weakest. In comparison to OLR and GWR models, the MGWR model, addressing the multiscale issue of regression coefficients, exhibited superior performance in local spatial regression.

In conclusion, this study provides valuable insights into the spatiotemporal dynamics and driving factors of LST in Jinan. The findings contribute to a more comprehensive understanding of the UHI effect, facilitating informed decision-making for sustainable urban development.
